# Efficacy of the Whole-Course Case Management Model on Compliance and Satisfaction of Breast Cancer Patients with Whole-Course Standardized Treatment

**DOI:** 10.1155/2022/2003324

**Published:** 2022-06-24

**Authors:** Lijun Jin, Yunyan Zhao, Peng Wang, Ran Zhu, Jie Bai, Jie Li, Xue Jia, Zunyi Wang

**Affiliations:** ^1^Department of Thyroid and Breast Surgery Department III, Cangzhou Central Hospotal, Cangzhou, China; ^2^Department of Tumour Medicine Ward, Cangzhou Central Hospital, Cangzhou, China

## Abstract

**Objective:**

To explore the influence of the whole-course case management model on the compliance and satisfaction of breast cancer patients with the whole-course standardized treatment.

**Methods:**

Eighty breast cancer patients admitted to our hospital between April 2020 and June 2021 were assigned to receive either conventional nursing (routine group, *n* = 40) or whole-process case management (experimental group, *n* = 40) according to different nursing methods. Outcome measures included self-rating anxiety scale (SAS) scores, self-rating depression scale (SDS) scores, adverse reactions, treatment compliance, and nursing satisfaction.

**Results:**

After nursing, the SAS and SDS scores of the experimental group were significantly lower than those of the routine group (*P* < 0.05). The whole case management mode was associated with a significantly lower incidence of adverse reactions versus routine nursing (*P* < 0.05). The whole case management resulted in higher compliance of patients versus routine nursing (*P* < 0.05). The experimental group had a significantly higher nursing satisfaction versus the routine group (*P* < 0.05).

**Conclusion:**

The whole-process case management mitigates patients' negative emotions, strengthens their treatment compliance, lowers the incidence of postoperative adverse reactions, and improves nursing satisfaction, which may provide a viable nursing alternative for patients with breast cancer.

## 1. Introduction

Breast cancer is the most common female malignancy worldwide and shows inclination in the younger population, posing a tremendous threat to women's health [1]. Research has shown that 70% of breast cancer patients experience psychological problems, and 18–50% of them have reoccurrence 1 year postoperatively [2]. Psychological problems substantially compromise the treatment effect, prognosis, and quality of life [3]. Due to insufficient resources and uneven abilities of nursing staff, conventional nursing fails to meet the complex needs of long-term comprehensive treatment of breast cancer patients [4]. Therefore, individualized management tailored for varying patient conditions is needed [5]. The whole-process case management is an individualized management model that provides patients with comprehensive and continuous nursing services by integrating medical resources and providing dynamic, continuous, individualized, and whole-process professional guidance to patients [6, 7]. Currently, chemotherapy is a treatment modality for breast cancer patients, which can effectively inhibit the spread of cancer cells to other organs and tissues; however, chemotherapy is associated with adverse events and negative emotions of patients, which predispose to treatment discontinuation due to drug intolerance of patients, seriously compromising the treatment outcomes. Therefore, effective nursing interventions are essential to improve treatment adherence, reduce adverse effects of chemotherapy, and improve their quality of life. The use of whole-process case management in the nursing of breast cancer patients has been marginally explored. Here, the present study was conducted to explore the influence of the whole case management model on the compliance and satisfaction of breast cancer patients with standardized treatment.

## 2. Study Design and Methods

### 2.1. Patients

Eighty breast cancer patients admitted to our hospital between April 2020 and June 2021 were assigned to receive either conventional nursing (routine group, *n* = 40) or whole-process case management (experimental group, *n* = 40) according to different nursing methods. The research was approved by the Ethics Committee of the Cangzhou Central Hospital. The ethics certificate number is 2019-11-11.

### 2.2. Inclusion and Exclusion Criteria

#### 2.2.1. Inclusion Criteria

Patients who were diagnosed with breast cancer for the first time, met the criteria in the Guidelines and Specifications for the Diagnosis and Treatment of Breast Cancer by the Chinese Anti-Cancer Association, and with consent to the study were included.

#### 2.2.2. Exclusion Criteria

Patients with other major diseases, with other malignant tumors, with stage IV breast cancer, with disease recurrence or death during follow-up, with cognitive impairment or mental illness, and with serious cardiovascular disease and other comorbidities were excluded.

### 2.3. Treatments

(1) The patients in the routine group received routine nursing. Routine nursing included routine examinations, vital signs monitoring, timely medical intervention, perioperative care, and medication guidance.
(2) The patients in the experimental group received whole case management. Case management team establishment: a case management team was established with the team leader with more than 5 years of clinical experience as a breast cancer specialist and over 1 year of practical experience in case management. Implementation of case management: the whole case management was performed upon diagnosis, during treatment, and during follow-up.
  1. The whole process of case management was carried out as per the working procedures for breast cancer case management
  2. A case management file was established for each patient, including general information, disease information, physical and mental status, family income, family map, source of medical expenses, social support, and psychosocial adaptability. Individualized care plans were formulated, including the operation date, operation method, chemotherapy regimens, chemotherapy dose, chemotherapy duration, adverse reactions, psychological status, and treatment cooperation, to form a continuous dynamic medical record.
  3. The patients were given health education, and their disease conditions were closely monitored, such as wound healing, limb function, side effects of chemotherapy drugs, and vascular protection.
  4. The nursing plan was coordinated with the whole process of individualized diagnosis and treatment plan. The responsible nurses of each patient participated in weekly case discussion and multidisciplinary team discussion to facilitate referrals and resource integration based on patient condition. The patients were asked for feedback about the nursing and were given various forms of health education, including audio and video materials on breast cancer. Patients' questions were timely resolved. The discharge plan and drug management plan were formulated for patients to be discharged, and the patients were also given discharge instructions. Telephone follow-up was commenced after all chemotherapy courses were completed within one month after discharge. The symptoms of patients were timely obtained and evaluated for timely medical feedback.
  5. The patients' conditions were evaluated as per Breast Cancer Hope Care, including basic information, pathological classification, specialist treatment, and review records. The results were presented to patients in the form of face-to-face interviews before the completion of chemotherapy and before the follow-up.
  6. The patients' psychological status, especially their psychosocial adaptability, were assessed and given professional psychological guidance by psychotherapists to relieve their negative emotions, thereby enhancing their psychosocial adaptability and quality of life.
  7. Formulation of health education strategies: the patients were educated about the importance of physiological and psychological health education; patients undergoing surgery + radiotherapy + chemotherapy would face more challenges in terms of physiological and psychological health, and priorities of health education should be tilted to such patients, including dietary guidance, exercise instructions, and psychological guidance; multiformat health education on breast cancer should be publicized through media such as television, the Internet, newspapers, and magazines; and nursing staff received courses related to health education to understand the impact of different treatment modalities on breast cancer patients and to develop and implement health education that meets the physical and psychological needs of patients.

### 2.4. Outcomes

1. Mental state: the self-rating anxiety scale (SAS) [8] was used to evaluate the degree of anxiety of the patients, with a score of 0–100 points and a cutoff value of 50 points, of which 50–59 was considered mild anxiety, 60–69 was considered moderate anxiety, and more than 69 was considered severe anxiety. The self-rating depression scale (SDS) [8] was used to evaluate the degree of depression of the patients, with a score of 0–100 points and a cutoff value of 53 points, of which a score of 53–62 was considered mild depression, 63–72 was considered moderate depression, and 73 was considered severe depression.
2. Adverse reactions: adverse reactions include bleeding, incision infection, axillary lymphatic leakage, and loss of appetite.
3. Treatment compliance: Good: the patient cooperates with the treatment and daily care. Modest: the patient partially cooperates with the treatment and daily care. Poor: the patient does not cooperate with the treatment and daily care.
4. Nursing satisfaction: The Nursing Satisfaction Questionnaire made by the hospital was used for evaluation. There are 20 questions in total, and the patients score the nursing according to the satisfaction of the nursing content, with 5 points for each question. A score of <70 points is dissatisfied, 70–89 is satisfied, and ≥90 is highly satisfied. Satisfaction = (highly satisfied + satisfied)/total number of cases × 100%.

### 2.5. Statistical Analysis

SPSS 21.0 was used for data analyses. Measurement data are expressed as (mean ± SD) and analyzed using the independent samples *t*-test. Count data are expressed as number of cases (rate) and analyzed using the chi-square test. Statistical significance was assumed at *P* < 0.05. The graphics were plotted using GraphPad Prism8.

## 3. Results

### 3.1. Baseline Patient Profile

The average age of patients in the routine group was 47.60 ± 12.23 years, 2 cases unmarried, 38 cases married, and there were 4 cases of stage I, 28 cases of stage II, and 8 cases of stage III breast cancer. The average age of patients in the experimental group was 47.18 ± 12.17 years, 1 case unmarried, 39 cases married, and there were 3 cases of stage I, 27 cases of stage II, and 10 cases of stage III breast cancer. The baseline data were comparable between the two groups (Table 1).

### 3.2. SAS and SDS Scores

Before nursing, the SAS and SDS scores were similar in the two groups (*P* > 0.05). After nursing, the SAS and SDS scores of the experimental group were significantly lower than those of the routine group (*P* < 0.05, Table 2).

### 3.3. Adverse Reactions

The whole case management was associated with a significantly lower incidence of adverse reactions versus the routine nursing (*P* < 0.05, Table 3).

### 3.4. Compliance of Patients

The whole case management resulted in higher compliance versus routine nursing (*P* < 0.05, Table 4).

### 3.5. Nursing Satisfaction

The experimental group had a significantly higher nursing satisfaction versus the routine group (*P* < 0.05, Table 5).

## 4. Discussion

Breast cancer is a malignant tumor that occurs in the mammary epithelium or ductal epithelium. The etiology of the disease remains unclear, and its influencing factors such as family history, breast cancer-related genes, reproductive factors, sex hormones, nutrition and diet, and environmental factors have been identified [8]. Due to the insidious symptoms of early breast cancer, the disease easily progresses to an advanced stage with symptoms such as nipple retraction and axillary lymph node enlargement [9]. With the advancement of modern medical technology, the long-term survival of breast cancer patients has also been expanded. However, patients still face relapse, poor quality of life, and aesthetics and psychological issues [10]. The negative impact of breast cancer surgery on the psychology of patients cannot be ignored during nursing [11].

Brenes et al. [12] stated that timely and effective psychological counseling for breast cancer patients could help patients relieve their negative emotions, thereby significantly improving patients' treatment compliance [13]. The present study found that after nursing, the SAS and SDS scores of the patients in the experimental group were significantly lower than those in the routine group, which is indicative of the effectiveness of the whole-process case management model in relieving negative emotions of breast cancer patients, which is attributed to the positive effects of psychological interventions in the whole-process case management model [14]. Chai et al. [15] revealed that a positive and reasonable nursing program for breast cancer patients after surgery could effectively reduce the occurrence of adverse reactions in patients. In the present study, the results showed that the experimental group had a lower incidence of adverse reactions, suggesting that the whole case management model contributes to lower postoperative adverse reactions in breast cancer patients. It is presumably due to the fact that the one-to-one case management boosts the patient's self-confidence in disease management, thereby promoting the recovery of the patient's body function, improving the patient's self-efficacy and self-management behavior, and thus lowering the incidence of postoperative adverse reactions in cancer patients [16]. As previously noted, surgical trauma, disease recurrence, anxiety, and uncertainty of prognosis adversely affect the patients physiologically and psychologically, thereby compromising the patient's treatment compliance [17]. Here, the results revealed that the whole case management mode resulted in higher compliance versus the routine nursing intervention. The reason may be that case management integrates high-quality resources and provides patients with systematic nursing services via assessment, planning, implementation, evaluation, and feedback, thereby fulfilling the complex psychological and physical needs of patients [18]. As a result, the patient's treatment compliance improved [19]. Additionally, a higher nursing satisfaction of the patients in the experimental group than in the routine group in the present study suggested a recognized satisfaction of the whole case management model by the patients and their families [20].

Surgery is the least detrimental to the mental health of postoperative breast cancer patients, while surgery + chemotherapy + radiotherapy poses the greatest impact on the psychological well-being of breast cancer patients. Chemotherapy is an effective adjuvant treatment to reduce the recurrence of breast cancer, but its excessively long course of treatment is associated with adverse physical and psychological effects on patients, with symptoms such as fatigue, sleep disturbance, anxiety, and depression. These adverse reactions and the periodicity of chemotherapy result in agitation and annoyance in patients, thereby developing fear and depression about chemotherapy [9]. Therefore, nursing staff should understand the psychological condition of patients and carry out targeted multilevel health education activities with appropriate timing and methods to improve the effectiveness of health education.

## 5. Conclusion

The whole-process case management mitigates patients' negative emotions, strengthens their treatment compliance, lowers the incidence of postoperative adverse reactions, and improves nursing satisfaction, which may provide a viable nursing alternative for patients with breast cancer. The limitation of this study is the inclusion bias, and future randomized multicenter studies will be conducted with increased follow-up to obtain patients' disease-free survival to provide more reliable clinical data.

## Figures and Tables

**Table 1:** Patients' profile (*n* (%)).

	Routine group (*n* = 40)	Experimental group (*n* = 40)	*t*/*x*^2^	*P*
Mean age (year)	47.60 ± 12.23	47.18 ± 12.17	0.156	0.877
Staging			0.383	0.826
I	4 (10.0)	3 (7.5)		
II	28 (70.0)	27 (67.5)		
III	8 (20.0)	10 (25.0)		
Marital status			0.346	0.556
Unmarried	2 (5.0)	1 (2.5)		
Married	38 (95.0)	39 (97.5)		

**Table 2:** SAS and SDS (*x* ± *s*).

Groups	*n*	SAS (point)		SDS (point)	
		Before	After	Before	After
Routine group	40	68.73 ± 2.36	58.13 ± 2.44	65.38 ± 3.18	53.93 ± 5.78
Experimental group	40	68.58 ± 2.29	48.45 ± 2.68	65.50 ± 3.20	47.55 ± 5.42
*t*	—	0.288	16.883	−0.175	5.090
*P*	—	0.774	<0.001	0.861	<0.001

**Table 3:** Adverse reaction (*n* (%)).

Groups	*n*	Bleeding	Wound infection	Axillary lymphatic leakage	Loss of appetite	Total
Routine group	40	3 (7.5)	2 (5.0)	2 (5.0)	6 (15.0)	13 (32.5)
Experimental group	40	0 (0)	0 (0)	0 (0)	1 (2.5)	1 (2.5)
*x* ^2^	—	—	—	—	—	12.468
*P*	—	—	—	—	—	<0.001

**Table 4:** Treatment compliance (*n* (%)).

Groups	*n*	Good	Modest	Poor
Routine group	40	11 (27.5)	17 (42.5)	12 (30.0)
Experimental group	40	33 (82.5)	6 (15.0)	1 (2.5)
*t*	—	24.444	7.384	11.114
*P*	—	<0.001	0.007	0.001

**Table 5:** Satisfaction (*n* (%)).

Groups	*n*	Very satisfied	Satisfied	Unsatisfied	Total
Routine group	40	8	21	11	29 (72.5%)
Experimental group	40	34	6	0	40 (100%)
*t*	—	—	—	—	12.754
*P*	—	—	—	—	<0.001

## Data Availability

The datasets used during the present study are available from the corresponding author upon request.
